# Take-home naloxone programs for suspected opioid overdose in community settings: a scoping umbrella review

**DOI:** 10.1186/s12889-021-10497-2

**Published:** 2021-03-26

**Authors:** Amina Moustaqim-Barrette, Damon Dhillon, Justin Ng, Kristen Sundvick, Farihah Ali, Tara Elton-Marshall, Pamela Leece, Katherine Rittenbach, Max Ferguson, Jane A. Buxton

**Affiliations:** 1 BC Centre for Disease Control, Vancouver, BC Canada; 2 Faculty of Medicine, University of British Columbia, Vancouver, BC Canada; 3 Institute for Mental Health Policy Research, Centre for Addiction and Mental Health (CAMH), London, ON Canada; 4 Department of Epidemiology and Biostatistics, Western University, London, ON Canada; 5 Dalla Lana School of Public Health, University of Toronto, Toronto, ON Canada; 6 Public Health Ontario (PHO), Toronto, ON Canada; 7 Alberta Health Services (AHS), Edmonton, AB Canada; 8 University of Alberta, Edmonton, AB Canada; 9 University of Calgary, Calgary, AB Canada; 10 School of Population and Public Health, University of British Columbia, Vancouver, BC Canada

**Keywords:** Naloxone, Opioid overdose, Fentanyl, Opioids, Opiates

## Abstract

**Background:**

Opioid related overdoses and overdose deaths continue to constitute an urgent public health crisis. The implementation of naloxone programs, such as ‘take-home naloxone’ (THN), has emerged as a key intervention in reducing opioid overdose deaths. These programs aim to train individuals at risk of witnessing or experiencing an opioid overdose to recognize an opioid overdose and respond with naloxone. Naloxone effectively reverses opioid overdoses on a physiological level; however, there are outstanding questions on community THN program effectiveness (adverse events, dosing requirements, dose-response between routes of administration) and implementation (accessibility, availability, and affordability). The objective of this scoping review is to identify existing systematic reviews and best practice guidelines relevant to clinical and operational guidance on the distribution of THN.

**Methods:**

Using the Arksey & O’Malley framework for scoping reviews, we searched both academic literature and grey literature databases using keywords (Naloxone) AND (Overdose) AND (Guideline OR Review OR Recommendation OR Toolkit). Only documents which had a structured review of evidence and/or provided summaries or recommendations based on evidence were included (systematic reviews, meta-analyses, scoping reviews, short-cut or rapid reviews, practice/clinical guidelines, and reports). Data were extracted from selected evidence in two key areas: (1) study identifiers; and (2) methodological characteristics.

**Results:**

A total of 47 articles met inclusion criteria: 20 systematic reviews; 10 grey literature articles; 8 short-cut or rapid reviews; 4 scoping reviews; and 5 other review types (e.g. mapping review and comprehensive reviews). The most common subject themes were: naloxone effectiveness, safety, provision feasibility/acceptability of naloxone distribution, dosing and routes of administration, overdose response after naloxone administration, cost-effectiveness, naloxone training and education, and recommendations for policy, practice and gaps in knowledge.

**Conclusions:**

Several recent systematic reviews address the effectiveness of take-home naloxone programs, naloxone dosing/route of administration, and naloxone provision models. Gaps remain in the evidence around evaluating cost-effectiveness, training parameters and strategies, and adverse events following naloxone administration. As THN programs continue to expand in response to opioid overdose deaths, this review will contribute to understanding the evidence base for policy and THN program development and expansion.

## Introduction

Opioid related overdoses and overdose deaths continue to present an urgent public health crisis worldwide. The World Health Organisation (WHO) estimates 115,000 people died from opioid overdose in 2017 [1]. In Canada, 16,364 people died of opioid-related overdoses between January 2016 and March 2020 [2], and the number of opioid-related deaths continues to rise [2]. In 2017, 46,802 drug overdose deaths in the United States involved opioids [3]. Spikes in opioid overdose deaths are related to both prescription opioids and to the emergence of the synthetic opioid fentanyl and its analogues in the unregulated market [4, 5]. Fentanyl is marked by high lipid solubility, leading to faster penetration of the blood-brain barrier and rapid respiratory depression compared to other opioids [6], necessitating rapid response in cases of overdose. The distribution of naloxone through programs colloquially referred to as ‘take-home naloxone’ (THN) has emerged as a key intervention to reduce opioid overdose deaths.

Opioids are substances derived from the opium poppy (natural opiates) or chemically synthesized, and often used or prescribed for pain [1]. Naloxone is a μ-opioid receptor antagonist effective at temporarily reversing the symptoms of opioid toxicity and life-threatening respiratory depression [7]. While naloxone has been used reliably in hospital settings to reverse opioid overdoses for over 50 years [8], the advent of THN programs and naloxone distribution and use by community members has expanded widely in more recent years. The first community-based naloxone projects in the United States and Europe started in the 1990s [9–11]. Given the dramatic increase in fatal opioid overdoses over the past decade in the United States and Canada, these jurisdictions began to prioritise increased access to naloxone and overdose education. In many countries worldwide, naloxone is only available to health professionals, although some jurisdictions are adopting policies to make the antidote more widely accessible [1]. Currently, naloxone is available in pharmacies without prescription in Australia, Canada, Italy, the United Kingdom of Great Britain and Northern Ireland, and Ukraine [1]. In Canada, two provincial programs (British Columbia and Ontario) were introduced before 2015, with the remaining eleven provinces and territories introducing programs between 2015 and 2018 [12]. As of December 2018, more than 590,000 naloxone kits had been distributed across Canada [2]. Preliminary evidence suggests that THN has helped avert thousands of additional opioid overdose deaths [13, 14].

Generally, THN programs aim to equip individuals who are at risk of witnessing or experiencing an opioid overdose with naloxone and to train them in overdose recognition and response. In Canada, THN kits generally include a carrying case, non-latex gloves, alcohol swabs, a face shield for providing rescue breaths, instructions on overdose response, and either injectable or nasal formulations of naloxone, depending on the province [12]. Preliminary evidence suggests that THN has been an effective intervention at preventing opioid overdose deaths [9, 14, 15]. However, there are outstanding questions regarding THN program effectiveness and implementation, including adverse events after naloxone administration, naloxone dosing requirements and dose-response between routes of administration, and access (including accessibility, availability, and affordability).

We conducted an umbrella scoping review (review of reviews) of the literature to characterise the existing knowledge base related to the use of naloxone for reversal of opioid overdose. The current paper will help identify gaps in the current evidence needed to inform clinical and operational guidance. Up-to-date guidance is critically needed to assist healthcare providers, policy makers, and program administrators in decisions regarding naloxone access, use, distribution, and training of bystanders. The results from this review can similarly be applied to understand the scope of knowledge relevant to standards for naloxone distribution and administration in other jurisdictions.

## Methods

### Design

The umbrella scoping review was conducted in adherence with the Arksey & O’Malley framework for scoping reviews [16]. Updates to this original framework by Levac et al. [17] were used to guide the methodology of this scoping review. Findings are reported in accordance with the Preferred Reporting Items for Systematic Reviews and Meta-Analyses (PRISMA) checklist for Scoping Reviews guidelines [18].

### Eligibility criteria

We confined our search to sources that described the use of naloxone for opioid overdose events, in any context that could reasonably relate to its distribution in the community for use by members of the general public. We included documents that had a structured review of evidence and/or provided summaries or recommendations based on evidence. This included systematic reviews, meta-analyses, scoping reviews, short or rapid reviews, practice guidelines, clinical guidelines, various reports, and working papers. We did not limit our search by timeframe – all databases of published literature were searched from database inception date to present. Sources were limited to those published or translated into English or French.

Due to the variability in the comprehensiveness and objectiveness of analysis in narrative reviews, these were excluded. Grey literature sources were limited to those published by a government (municipal, provincial or federal level), non-profit organisation, academic organisation, or professional medical society – documents published by private businesses or industry were excluded. No exclusions were made based on geographic location.

### Information sources

A search strategy was developed and refined with the help of a research librarian. Academic literature databases and grey literature databases were searched. We searched the following databases for peer-reviewed literature: Ovid Medline, Embase, the Cumulative Index to Nursing and Allied Health Literature (CINAHL), PsycINFO, Prospero, and Epistemonikos.

We defined grey literature as literature not published in books or journals [19]. The process outlined by Godin et al. [20] was used to identify evidence from the grey literature. This process incorporates four different searching strategies: 1) Searching grey literature databases, 2) using Customized Google searches, 3) searching targeted websites, and 4) consultation with content experts. Grey literature databases included Guidelines International Network (GIN), Open Grey: System for Information on Grey Literature of Medicine, and Grey Literature Report. Customized Google searches were performed, and the first 100 hits evaluated. Targeted websites included government websites in Canada, the United States, Europe, and Australia, reflecting regions affected by the opioid crisis related to regional drug supply [21]. Non-governmental and think tank websites including the Bill and Melinda Gates Foundation, WHO, United Nations, Canadian Centre on Substance Use and Addiction (CCSA) and the Canadian Agency for Drugs and Technology in Health (CADTH) were also searched. A list of content experts was developed and a request for referred literature and projects in progress was sent, with follow up at two weeks.

### Search

The following search terms were used and modified, if necessary, for the search: (Naloxone) AND (Overdose) AND (Guideline OR Review OR Recommendation OR Toolkit). Searches were performed from database inception to April 2020 and updated in June 2020. See Table 1 for an example search strategy in Ovid Medline.

**Table 1:** Example search strategy - Medline Ovid

Database(s): Ovid MEDLINE(R) and Epub Ahead of Print, In-Process & Other Non-Indexed Citations, Daily and Versions(R) 1946 to April 03, 2020		
#	Searches	Results
1	Naloxone/	18,648
2	(naloxon* or narcan*).mp. [mp = title, abstract, original title, name of substance word, subject heading word, floating sub-heading word, keyword heading word, organism supplementary concept word, protocol supplementary concept word, rare disease supplementary concept word, unique identifier, synonyms]	27,182
3	Drug Overdose/	11,165
4	overdos*.mp. [mp = title, abstract, original title, name of substance word, subject heading word, floating sub-heading word, keyword heading word, organism supplementary concept word, protocol supplementary concept word, rare disease supplementary concept word, unique identifier, synonyms]	24,133
5	1 or 2	27,182
6	3 or 4	24,133
7	5 and 6	1550
8	limit 7 to (english or french)	1527

Searching the grey literature involves using databases with a wide variance in search functionalities and filters available for retrieving results. As such, search terms were adapted to fit each database and its usability.

### Selection of evidence

All of the search results were exported into the reference manager Zotero [22], and then added to the systematic review software Covidence [23]. Duplicates were identified and removed. In cases where reports or evidence reviews were updates of previous reports or reviews, only the most recent version was included. Two reviewers (AMB and JN) independently screened published articles based on information contained in the title, abstracts, and key words. For any uncertainties or disagreements, articles were discussed by both reviewers until agreement was reached. For grey literature searches, one reviewer (AMB) reviewed the title and summary lines from each entry for relevance. Full grey literature reviews were then conducted by the same two independent reviewers (AMB and JN), and reasons for exclusion were recorded. Where full documents could not be accessed, our team contacted the authors with a request for the document or an update on the status of the title. Conflicts were again re-evaluated by both reviewers and each resolved through discussion. The reference lists of included articles were then checked (citation chained). In cases where Covidence failed to remove duplicates, duplicates were removed during full text screening. Quality appraisal was not performed or used for study selection.

### Data extraction

Data were extracted using a structured data abstraction form designed in REDCap, a web-based data collection tool that allows users to build and manage databases [24]. The abstraction form was first piloted by four independent reviewers (AMB, JN, DD, and KS) using a total of three selected articles each, and revisions were made through consensus discussion. Three key areas were used for extraction: [25] Study identifiers (article title; journal title; authors; country of the study; language; publication year) [1]; methodological characteristics (study design; study objective, research question, or hypothesis; study population; data sources; statistical analyses) [2]; main outcomes measured. Some articles constituted larger reviews of harm reduction interventions. For all articles, the number of primary studies specifically related to naloxone was extracted. Of these, the number of randomized controlled trials (RCTs) evaluated by the articles was also examined.

Data were extracted by authors AMB and JN and validated by authors KS and DD. Any conflicts were resolved through discussion. Once finalised, data from REDCap was exported, cleaned, and analysed using R version 3.5.3 [26].

## Results

### Overview

**Fig. 1:**
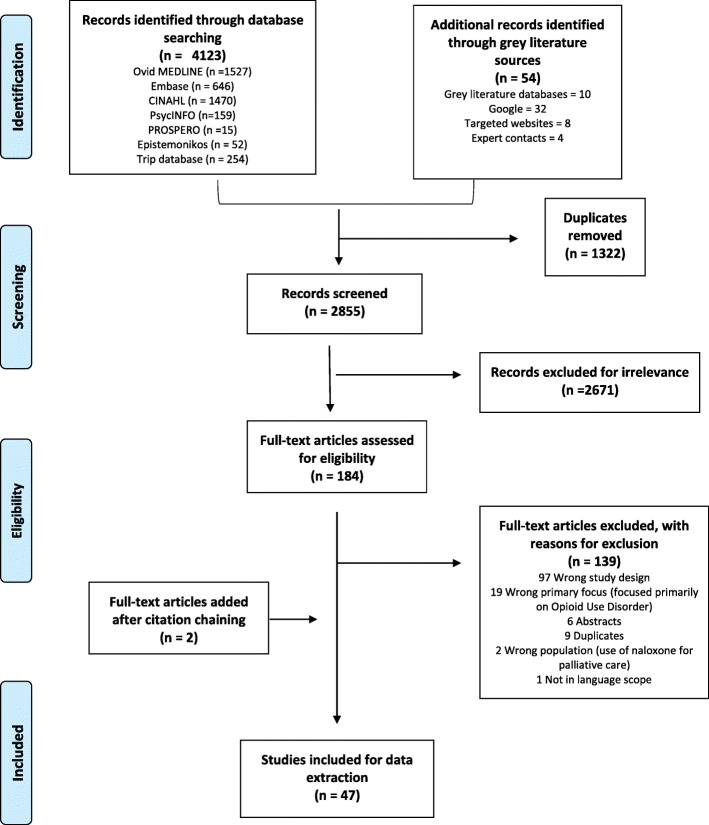
PRISMA (Preferred Reporting Items for Systematic Reviews and Meta-Analyses) Flow Diagram

A total of 127 articles underwent full-text review, and 47 unique articles ultimately met the inclusion criteria - see Fig. 1 for a PRISMA flow diagram on evidence selection. This review sought to identify evidence syntheses which used systematic methods to identify primary research. As such, no primary research articles were included in the review. The most common reason for exclusion was that the study did not provide structured review of evidence and/or did not provide summaries or recommendations based on evidence- most often excluded studies were narrative reviews.

Methodological characteristics of the articles included can be found in Table 2 and Table 3. A total of 20 systematic reviews were identified, 10 evidence syntheses from the grey literature, 8 ‘short-cut’ or rapid reviews, 4 scoping reviews, and 5 other reviews (e.g. mapping review and comprehensive reviews) types. Of the systematic reviews, five articles used meta-analyses, and 16 articles examined results from Randomized Controlled Trials (RCTs). During data extraction, all articles were categorised by reviewers into larger subject themes. Table 4 provides included literature by subject theme. The subject themes which arose most frequently were: naloxone dosing and routes of administration (*n* = 14, 29.8%), provision, feasibility, and acceptability of naloxone distribution (*n* = 13, 27.7%), effectiveness of naloxone and take-home naloxone for opioid overdose reversal (*n* = 10, 21.3%), overdose response after naloxone administration (*n* = 6, 12.8%), naloxone training and education (n = 6, 12.8%), recommendations for policy, practice and gaps in knowledge (*n* = 4, 8.5%), naloxone safety (harms or adverse events related to naloxone administration) (*n* = 3, 6.4%), and cost-effectiveness (n = 3, 6.4%).

**Table 2:** Methodological characteristics of included systematic reviews

Document	Document Type	Stated purpose	Themes	Studies reviewed related to naloxone	Year of last study reviewed	RCTs	Meta-analysis
Bahji_2018 [27]	Systematic review	To synthesize findings and provide a systematic review of interventions for the treatment and prevention of opioid overdose.	Effectiveness of naloxone and take-home naloxone (THN) for opioid overdose reversal	4	2016	12	No
Behar_2018 [28]	Systematic review	To assess the acceptability and feasibility of prescribing naloxone to patients in primary care settings.	Provision, feasibility and acceptability of naloxone distribution	17	2017	–	No
Chimbar_2018 [29]	Systematic review	To examine the effectiveness of naloxone take-home kits and their effect in reducing fatal overdoses among those who use opioids.	Effectiveness of naloxone and take-home naloxone (THN) for opioid overdose reversal	9	2016	2	No
Chou_2017 [30]	Systematic review	To synthesize evidence on 1) the effects of naloxone route of administration and dosing for suspected opioid overdose in out-of-hospital settings on mortality, reversal of overdose, and harms, and 2) the need for transport to a health care facility after reversal of overdose with naloxone.	Naloxone dosing or routes of administration Overdose response after naloxone administration	13	2016	3	No
Clark_2014 [31]	Systematic review	To describe the current state of the literature on community-based opioid overdose programs (OOPPs) with a focus on the effectiveness of these programs. This article reviews characteristics and outcomes of OOPPs.	Effectiveness of naloxone and take-home naloxone (THN) for opioid overdose reversal Naloxone training and education	19	2013	–	No
Eizadi-Mood_2019 [32]	Systematic review	To examine the relationship between naloxone and seizure in tramadol poisoning.	Naloxone safety (harms or adverse events related to naloxone administration)	7	2015	1	Yes
EMCDDA_2015 [11]	Systematic review	To assess the effect of take-home emergency naloxone and educational intervention on knowledge improvement, naloxone use, management of overdoses witnessed and death as a result of overdose.	Effectiveness of naloxone and take-home naloxone (THN) for opioid overdose reversal Naloxone training and education	21	2014	1	No
Giglio_2015 [33]	Systematic review	To synthesize the quantitative findings of available studies to generate a summary estimate of the effectiveness of bystander naloxone administration and overdose education programs using meta-analytic methods.	Effectiveness of naloxone and take-home naloxone (THN) for opioid overdose reversal Naloxone training and education	9	2014	1	Yes
Greene_2019 [34]	Systematic review	To determine mortality and serious adverse events within 48 h of EMS treat and release due to suspected rebound opioid toxicity after naloxone administration.	Overdose response after naloxone administration	7	2017	–	Yes
Gunn_2018 [35]	Systematic review	To assess the effectiveness of the Emergency Department as a potential setting for naloxone distribution for overdose reversal. The purpose of this systematic review was to identify, evaluate, and summarize available evidence regarding the distribution of take-home naloxone in the ED and identify the areas that require future research.	Provision, feasibility and acceptability of naloxone distribution	5	2016	1	No
Haegerich_2019 [36]	Systematic review	To assess systems-level interventions to address provider and patient/public behavior and prevent prescription and illicit opioid overdose, naloxone education and distribution	Provision, feasibility and acceptability of naloxone distribution	65	2019	1	No
McAuley_2015 [37]	Systematic review	To assess the impact of take-home naloxone at a global level, in order to give effect sizes that could be extrapolated to different populations across the world.	Provision, feasibility and acceptability of naloxone distribution	9	2012	9	Yes
McDonald_2016 [9]	Systematic review	To assess the effectiveness of take-home naloxone (THN), addressing the following two aims: [25] to describe the impact of THN provision on overdose related mortality in opioid users; and [1] to assess the safety of THN provision by quantifying adverse events associated with naloxone administration.	Effectiveness of naloxone and take-home naloxone (THN) for opioid overdose reversal Naloxone safety (harms or adverse events related to naloxone administration) Cost-effectiveness	–	2015	–	No
Mitchell _2016 [38]	Systematic review	To identify trends in the current literature, gaps in the findings, nursing implications, and opportunities for further exploration related to the use of naloxone in opioid overdose.	Effectiveness of naloxone and take-home naloxone (THN) for opioid overdose reversal	8	2015	7	No
Moe_2020 [39]	Systematic review	To evaluate the relationship between naloxone dose (initial and cumulative) and opioid toxicity reversal and adverse events in undifferentiated and presumed fentanyl/ ultra-potent opioid overdoses.	Naloxone dosing or routes of administration	–	No date	7	No
Ryan_2018 [40]	Systematic review	To assess the pharmacokinetic properties of community-use formulations of naloxone for emergency treatment of opioid overdose.	Naloxone dosing or routes of administration	7	2017	7	No
Smart_2020 [41]	Systematic review	The review describes demographic and clinical characteristics of opioid overdose prevention program (OOPP) participants, describes OOPP curriculums and addresses the following questions: [25] Do OOPPs with naloxone distribution reduce fatal and nonfatal overdose rates among participants? [1] Are OOPPs effective at increasing nonmedical bystander knowledge of prevention, risk factors, and recognition of opioid overdose? [2] Do nonmedical bystanders trained at OOPPs respond correctly to witnessed opioid overdoses?	Provision, feasibility and acceptability of naloxone distribution	11	2019	–	No
Strang_2016 [42]	Systematic review	To examine the options for non-injectable naloxone with potential application for wider community-based opioid overdose reversal.	Naloxone dosing or routes of administration	21	2015	–	No
Thakur_2020 [43]	Systematic review	To examine the current state of naloxone use and dispensing regarding [25] roles for pharmacists dispensing naloxone, [1] barriers to their dispensing naloxone, and [2] pharmacist training to dispense naloxone.	Provision, feasibility and acceptability of naloxone distribution	33	2018	–	No
Yousefifard_2019 [44]	Systematic review	To compare the efficacy of the intranasal administration of naloxone with its intramuscular/intravenous administration in the pre hospital management of opioid overdose.	Naloxone dosing or routes of administration	6	2014	3	Yes

**Table 3:** Methodological characteristics of other studies – scoping reviews, short/cut rapid reviews, and reports

Document	Document type	Purpose	Topic	Studies reviewed related to naloxone	Year last study reviewed	Total RCTs
Bagley_2019 [45]	Scoping review	To identify US-based post-overdose intervention models described in peer-reviewed literature and implemented in public health and community settings	Effectiveness of naloxone and take-home naloxone (THN) for opioid overdose reversal	31	2018	–
Mauri_2020 [46]	Scoping review	To synthesize the available evidence on the effectiveness of prevalent state opioid policies on improving outcomes related to opioid prescribing and dispensing, patient behavior, and patient health	Provision, feasibility and acceptability of naloxone distribution	2	2018	2
Muzyk_2019 [47]	Scoping review	To identify, evaluate, and summarize published literature describing pharmacists’ attitudes toward naloxone and medications for opioid use disorder	Provision, feasibility and acceptability of naloxone distribution Naloxone training and education	5	2017	–
Nielsen_2016 [48]	Scoping review	To understand what is currently known about community pharmacy supply of naloxone, with a particular focus on understanding current practice and supply models, and barriers that may need to be addressed in order to embed and optimize the expansion of naloxone supply through this community route.	Provision, feasibility and acceptability of naloxone distribution	16	2016	–
Ashton_2006 [49]	Short cut/rapid review	To establish whether intranasal naloxone is effective in suspected opiate overdose.	Naloxone dosing or routes of administration	8	2005	3
Barrie_2006 [50]	Short cut/rapid review	To establish whether naloxone may have an ‘awakening effect’ in patients who have not taken opiates, thereby clouding its use as a diagnostic manoeuvre.	Naloxone safety (harms or adverse events related to naloxone administration)	3	1999	1
Barrie_2008 [51]	Short cut/rapid review	To establish whether the training of intravenous drug users in the use of naloxone and the prescription of that drug to those users reduces mortality from opiate overdose.	Effectiveness of naloxone and take-home naloxone (THN) for opioid overdose reversal	3	2006	–
Brooker_2019 [52]	Short cut/rapid review	To explore the experience in administering naloxone in a home or community setting by community and lay users, community service staff, police and other non-healthcare professionals, as well as allied health professional.	Provision, feasibility and acceptability of naloxone distribution	11	2019	–
Clarke_2002 [53]	Short cut/rapid review	To establish whether patients with no recurrence of symptoms one hour after receiving naloxone for an opioid overdose can safely be discharged.	Overdose response after naloxone administration	5	2000	–
Ishiyama_2013 [54]	Short cut/rapid review	To establish whether nebulised naloxone is a safe and effective alternative to intravenous naloxone in reversing opioid toxicity.	Naloxone dosing or routes of administration	2	2013	–
Kerr_2008 [55]	Short cut/rapid review	To review the effectiveness, safety and utility of intranasal naloxone for the treatment of heroin overdose.	Naloxone dosing or routes of administration	8	2005	2
Marshall_2018 [56]	Short cut/rapid review	To establish psychological impacts of being a peer-helper in a take home naloxone program	Provision, feasibility and acceptability of naloxone distribution	27	2015	4
Kampman_2015 [57]	Practice guidelines	To provide information on evidence-based treatment of opioid use disorder	Recommendations for policy and practice and gaps in knowledge	Unknown	No date	–
Strike_2015 [58]	Practice guidelines	To evaluate the effectiveness of harm reduction programs that deliver prevention services to people who use drugs and are at risk for human immunodeficiency virus (HIV), hepatitis C (HCV), hepatitis B (HBV), and other harms.	Recommendations for policy and practice and gaps in knowledge	22	2013	–
WHO_2014 [59]	Practice guidelines	To provide evidence-based recommendations on the availability of naloxone for people likely to witness an opioid overdose along with advice on the resuscitation and post-resuscitation care of opioid overdose in the community.	Recommendations for policy and practice and gaps in knowledge	3	2009	3
Williams_2019 [60]	Clinical practice guidelines	To develop and disseminate an evidence-based guideline and model protocol for administration of naloxone by EMS practitioners to persons with suspected opioid overdose.	Recommendations for policy and practice and gaps in knowledge	13	2016	3
CADTH_2007 [61]	Report	1) To evaluate the clinical benefit and harm of pre-hospital use of naloxone in adult patients with opiate overdose, 2) to evaluate the clinical evidence of the different routes of administering naloxone in adult patients with opiate overdose (in a pre-hospital setting), and 3) to evaluate existing guidelines for pre-hospital administration of naloxone to adult patients with opiate overdose.	Effectiveness of naloxone and take-home naloxone (THN) for opioid overdose reversal Naloxone dosing or routes of administration	10	2007	2
CADTH_2017 [62]	Report	To provide evidence on the comparative clinical effectiveness and cost effectiveness of the various formulations and delivery mechanisms of naloxone for the treatment of opioid poisoning.	Naloxone dosing or routes of administration	3	2015	2
CADTH_2014 [63]	Report	To determine the comparative clinical effectiveness of intranasal (IN) versus intravenous (IV) naloxone for treatment of suspected or apparent opioid overdose in the pre-hospital setting.	Naloxone dosing or routes of administration	2	2010	–
CADTH_2019 [64]	Report	To investigate 1) the clinical effectiveness of naloxone administered in a community or home setting and 2) The cost-effectiveness of naloxone administered in a home or community setting.	Effectiveness of naloxone and take-home naloxone (THN) for opioid overdose reversal Cost-effectiveness	6	2018	1
Lobmaier_2020 [65]	Report	To review interventions for non-fatal overdoses in order to make recommendations from the literature on a standardized patient pathway, especially as it relates to post-opioid overdose interventions.	Overdose response after naloxone administration	5	2019	–
PHO_2016 [66]	Report	To determine the effectiveness of rescue breathing only, conventional CPR, or neither by adult laypersons on survival in suspected opioid-associated resuscitation emergencies among adults in the community, compared to compression-only CPR used with or without naloxone.	Overdose response after naloxone administration	17	2016	–
Horton_2017 [67]	Mapping review	To map research into take home naloxone for people released from correctional settings in order to identify further research needs.	Provision, feasibility and acceptability of naloxone distribution	19	2016	3
Mueller_2015 [68]	Review	To classify existing publications on overdose education and naloxone distribution (OEND) programs and naloxone in community-based settings.	Provision, feasibility and acceptability of naloxone distribution Naloxone dosing or routes of administration Cost-effectiveness	41	No date	2
McDonald_2018 [69]	Review	To examine published international patent applications of non-injectable naloxone formulations and contributory pharmacokinetic (PK) data. Three aims: 1) to trace the concept and product development by route of administration; 2) to describe the non-injectable naloxone formulations for which human in vivo data are available; and 3) to compare human PK data reported in the patent applications.	Naloxone dosing or routes of administration	8	2015	–
Weaver_2018 [70]	Review	To investigate the various routes of naloxone administration for opioid reversal in the prehospital setting	Naloxone dosing or routes of administration	8	2015	2
Willman_2016 [71]	Review	To search the medical literature related to the following questions: [25] What are the medical risks to a heroin user who refuses ambulance transport after naloxone? [1] If the heroin user is treated in the emergency department with naloxone, how long must they be observed prior to discharge? [2] How effective in heroin users is naloxone administered by first responders and bystanders? Are there risks associated with naloxone distribution programs?	Overdose response after naloxone administration	29	2016	–

**Table 4:** Included literature by subject theme

Subject themes	Number of studies included (%)*	Studies included
Naloxone dosing or routes of administration	14 (29.8%)	[30, 39, 40, 42, 44, 49, 54, 55, 61–63, 68–70]
Provision, feasibility and acceptability of naloxone distribution	13 (27.7%)	[28, 35–37, 41, 43, 46–48, 52, 56, 67, 68]
Effectiveness of naloxone and take-home naloxone (THN) for opioid overdose reversal	10 (21.3%)	[9, 11, 27, 29, 31, 33, 38, 45, 51, 61, 64]
Overdose response after naloxone administration	6 (12.8%)	[30, 34, 53, 65, 66, 71]
Naloxone training and education	6 (12.8%)	[11, 31, 33, 36, 47, 51]
Recommendations for policy, practice, and gaps in knowledge	4 (8.5%)	[57–60]
Naloxone safety (harm and adverse events related to naloxone administration)	3 (6.4%)	[9, 32, 50]
Cost-effectiveness	3 (6.4%)	[9, 64, 68]

*Percentages do not add up to 100% because some document subject themes overlap

**Fig. 2:**
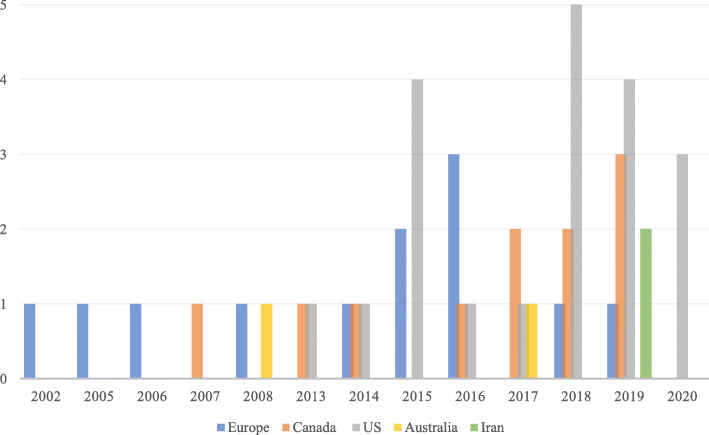
Histogram of region of origin and date of published naloxone research syntheses

Figure 2 presents the distribution of included articles according to year of publication and geographic location of origin. A total of 12 articles originated from Europe, 11 from Canada, 20 from the United States, two from Australia, and two from Iran. Reflecting the historical emergence of THN programs across jurisdictions, the earliest evidence syntheses emerged from Europe in early 2000. From 2015 to 2020, there was a notable increase in the number of articles addressing the use of naloxone in opioid overdose, with 38 evidence syntheses (80.6%) published in the last five years.

### Naloxone and THN program effectiveness in treatment of opioid overdose

Ten systematic reviews examined the effectiveness of naloxone or THN programs for the treatment of opioid overdose [9, 11, 27, 29, 31, 33, 37, 38, 45, 51, 61, 64]. Bahji et al. was the only systematic review to examine the effectiveness of naloxone as a candidate drug for opioid overdose reversal [27]. Another two reports sought to examine the clinical effectiveness of naloxone administered in pre-hospital, community, or home settings [61, 64].

A systematic review by McDonald and Strang [9] investigated the relationship between THN programs and opioid overdose mortality using Bradford-Hill criteria (minimal criterion for establishing causal inference). Several studies investigated the association between naloxone distribution and overdose reversal [31, 38] or a reduction in overdose mortality [11, 29].

One article used meta-analyses to generate an estimate of the effectiveness of bystander naloxone administration and overdose education programs on overdose recovery across nine primary articles [33]. One rapid review was carried out to establish whether the training of people who use intravenous drugs in the use of naloxone reduces mortality from opioid overdose [51].

### Provision, feasibility, and acceptability of naloxone distribution

Of the 13 articles that evaluated outcomes related to the provision, feasibility, and acceptability theme, several reviews evaluated naloxone provision in a specific setting. Thakur et al. performed a systematic review examining pharmacy dispensing and distribution of naloxone [43], while Muzyk et al. [47] and Nielsen et al. [48] performed scoping reviews related to pharmacy naloxone provision and pharmacist attitudes. Gunn et al. assessed distribution of naloxone from emergency departments [35], and Behar et al. assessed acceptability and feasibility of naloxone prescribing to patients in primary care settings [28]. One mapping review assessed evidence on THN distribution from correctional facilities to identify further research needs [67], and two rapid reviews assessed attitudes and experiences related to naloxone administration by community and lay users, service staff, police and other non-healthcare professionals [52, 56].

A review by Haegerich et al. [36] examined available evidence related to naloxone delivery modalities including 1) state legislation and regulation, 2) prescription drug monitoring programs (PDMPs), 3) insurance strategies, 4) clinical guideline implementation, 5) provider education, 6) health system interventions, 7) naloxone education and distribution, 8) safe storage and disposal, 9) public education, 10) community coalitions, and 11) interventions employing public safety and public health collaborations.

Mueller et al. [68] also sought to understand the extent of available evidence related to provider willingness to prescribe naloxone as well as experiences and attitudes of potential bystanders sampled from service users of harm reduction programs.

Two studies looked at drug policy within the United States; a systematic review [41] investigated the association of naloxone access laws and naloxone prescribing and distribution and a scoping review [46] identified literature on legislative and administrative policy interventions that evaluated prescribing and dispensing, patient behaviour, or patient health. Studies related to naloxone access laws were included in this theme given their effect on delivery modalities and acceptability from the perspective of various stakeholders.

Finally, a systematic review by McAuley et al. [37] sought to understand what proportion of distributed naloxone is used to respond to overdose in order to inform naloxone supply needs. The authors used meta-analyses to estimate what proportion of those trained and supplied with naloxone will use it within a given time period.

### Naloxone dosing and route of administration

We identified five systematic reviews focused on comparing the effectiveness between injectable (e.g., intravenous, subcutaneous, intramuscular) and non-injectable (e.g., intranasal, buccal, sublingual) naloxone routes of administration [30, 39, 40, 42, 44]. A final systematic review published in 2020 aimed to evaluate sufficient naloxone doses during an era of ultra-potent synthetic opioid use [39].

Another six non-systematic reviews examined topics related to routes of administration for opioid reversal. One review evaluated implications of different routes of administration for pharmacy practice (e.g., reasons for preferences) [70]. Another ‘comprehensive review’ performed an exploratory search of patent applications for non-injectable naloxone to expand knowledge on bioavailability of intravenous vs non-intravenous naloxone formulations [69]. The review by Mueller et al. also sought to identify evidence related to naloxone routes of administration, identifying a total of five controlled trials in pre-hospital settings comparing intranasal, intravenous, and intramuscular administration [68]. Three rapid reviews also aimed to identify evidence related to whether nebulized naloxone [54] and intranasal naloxone [49, 55] were effective alternatives to injectable formulations for overdose reversal.

The Canadian Agency for Drugs and Technologies in Health (CADTH) published three separate reports identified in the grey-literature comparing the clinical effectiveness of intranasal and intravenous naloxone for treatment of suspected opioid overdose [32, 49, 64].

### Naloxone safety – harms and adverse events related to naloxone administration

One identified systematic review with meta-analysis focused on potential harms after naloxone administration [32], specifically reviewing literature related to whether naloxone increased the risk of seizures after treatment of tramadol poisoning.

One rapid review aimed to establish evidence related to the effect of naloxone when used for patients with non-opioid toxicity. The review searched the literature to establish whether naloxone may have the same ‘awakening effect’ in patients with no reported recent opioid use [50].

A grey literature report by CADTH in 2017 reported on two unblinded randomized controlled articles comparing incidence of adverse events with naloxone administered intranasally using a mucosal atomizer and intramuscular naloxone, including agitation and/or aggression, nausea and/or vomiting, and headache [62].

### Overdose response following naloxone administration

Two systematic reviews examined the evidence related to the need for transport to hospital after naloxone administration, based on mortality or serious adverse events after treatment [30, 72]. One of the systematic reviews looked at naloxone administration by Emergency Medical Services (EMS) personnel, other health care providers, or laypersons [30] while the other looked exclusively at naloxone administration by EMS [72]. None of the primary articles compared outcomes between people transported and not transported to hospital [30]. Three more non-systematic review articles evaluated evidence related to the need for observation after treatment with naloxone [65, 73, 74]. A fourth evidence review examined the effectiveness of giving chest compression and/or rescue breaths after naloxone administration [66].

### Cost effectiveness

While we did not identify any systematic reviews focused specifically on cost-effectiveness of naloxone or naloxone distribution, two systematic reviews examined cost-effectiveness as secondary outcome measures [8, 45]. One of the two reported on separate modelling data from both the United States and Russia, and the other reported on the financial impact of intranasal naloxone compared to intramuscular forms [38]. Relying on the same two articles evaluated by McDonald et al., a review by Mueller et al. also examined the cost-effectiveness of naloxone distribution programs [68]. Two reports by CADTH attempted to synthesize evidence related to cost-effectiveness of naloxone distribution programs [62, 64].

### Naloxone education/training for bystanders

One systematic review attempted to quantify the effect of naloxone training programs based on overall average scores between trained participants compared with untrained participants on tests that covered overdose prevention material (naloxone administration, overdose recognition, overdose response) [33]. Two additional systematic reviews also synthesized evidence on naloxone training and education as secondary outcomes including improvement in knowledge immediately after training [10, 38],

### Recommendations for policy and practice and gaps in knowledge

Four practice guidelines were identified which used evidence syntheses to create recommendations for the use and/or distribution of naloxone. First, the World Health Organisation (WHO) published guidelines for community management of opioid overdose in 2014 [59]. For all key questions, the WHO assessed the quality of evidence based on Grading of Recommendations Assessment, Development and Evaluation (GRADE) criteria. Key questions included: 1) Should naloxone be distributed to people who are likely to witness an opioid overdose? 2) What formulation and dosage of naloxone should be used in the initial management of opioid overdose, including by lay responders, and in the pre-hospital setting? 3) Should the resuscitation response to suspected opioid overdose, including by layperson bystanders, be based on standard CPR or chest compression only CPR? 4) What should be the response to opioid overdose after the administration of naloxone and successful reversal of opioid overdose in the community, including by lay first responders?

In 2015, a Working Group on Best Practice for Harm Reduction Programs in Canada created recommendations for the use of naloxone in the event of an opioid overdose [58]. Additionally in 2015, the American Society of Addiction Medicine (ASAM) created a national practice guideline for the use of medications in the treatment of addiction involving opioid use, intended for clinicians involved in evaluating patients and providing authorization for pharmacological treatments at any level [57]. As it relates to the use of naloxone, the ASAM addressed naloxone administration in cases of opioid overdose (including for pregnant women), naloxone provision for patients being treated for opioid use disorder (OUD) and their families, and administration of naloxone by first responders. In 2019, Williams et al. published evidence-based guidelines for Emergency Medical Service (EMS) administration of naloxone [60], including route of administration.

### Discussion

This review scoped the existing literature for evidence syntheses related to the use and distribution of naloxone for reversal of opioid overdose in community settings. We identified a total of 47 articles, including 20 systematic reviews. We found that the majority of evidence syntheses related to naloxone evaluated the effectiveness of naloxone and THN programs in reducing opioid overdose mortality, examined optimal dosing or routes of administration for opioid overdose reversal, and documented barriers and facilitators to THN provision, feasibility and acceptability. Fewer evidence syntheses evaluated harms and adverse events related to naloxone administration, overdose response following naloxone administration, cost-effectiveness of naloxone distribution programs, and naloxone administration training strategies, and recommendations for policy and practice related to naloxone use and distribution.

While most review articles relied on observational data, there appears to be a variety of evidence addressing THN and overdose reversal and or overdose mortality. A number of systematic reviews have now also collated evidence related to available naloxone administration methods and optimal doses, both for contexts before and after the emergence of potent synthetic opioids (like fentanyl) on the illicit market, which may be used to inform naloxone provision and use.

Less of the evidence related to specific operational aspects or optimization of THN programs. Available distribution models, feasibility, and acceptability for naloxone distribution is dependent on jurisdiction and setting. For example, some provinces in Canada currently require pharmacist intervention for naloxone distribution, many jurisdictions in the United States require a prescription [45], while other provinces in Canada list naloxone as an unscheduled drug (drugs which can be sold without professional distribution) [12]. Given the different contexts and laws related to opioid and naloxone scheduling and availability [75], strategies related to distribution, feasibility, and acceptability will require jurisdiction-specific evidence.

In addition, only three studies examined evidence related to cost-effectiveness as secondary outcomes. Only one systematic review examined training parameters for naloxone administration, and one systematic review conducted, was related to adverse events following naloxone administration. Future evidence syntheses on these topics would help inform policy and practice.

The goal of this study was to identify gaps in the current evidence needed to inform clinical and operational guidance. Table 5 describes gaps in the literature by subject theme. While this study identified four best practice guideline recommendations published since 2014, three of these created recommendations that were intended for clinicians [57], EMS [60], or program administrators [58] rather than community members. In 2014, the World Health Organisation attempted to create best practice guidelines for community management of opioid overdose that would be applicable across jurisdictions, though recommendations relied on the scant evidence available at that time and should be updated [59].

**Table 5:** Identified research gaps by subject theme

Subject themes	Research Gaps
General	Updated comprehensive best practice guidelines for community management opioid overdose that would be applicable across jurisdictions [59]
Naloxone dosing or routes of administration	Optimal dosing and administration of naloxone given differing contexts and training
Provision, feasibility and acceptability of naloxone distribution	Jurisdiction-specific strategies related to distribution, feasibility, and acceptability, including distribution locations and eligibility
Overdose response after naloxone administration	Overdose response following naloxone administration, including identifying situations in need of emergency services, follow-up after overdose, and provision of resources and services for individuals who have overdosed.
Naloxone training and education	Training parameters and strategies, including identifying minimum training requirements and optimal learning strategies given the context and population
Recommendations for policy, practice, and gaps in knowledge	Recommendations for policy and practice related to naloxone use and distribution, including naloxone scheduling, pricing, and access Specific operational aspects and optimization of THN programs
Naloxone safety (harm and adverse events related to naloxone administration)	Adverse events and harms related to naloxone administration, including risk of withdrawal and other adverse outcomes, and effectiveness of
Cost-effectiveness	Cost-effectiveness of naloxone or naloxone distribution, including evaluating strategies for targeted vs general distribution

To our knowledge, this is the first scoping umbrella review conducted to examine evidence related to the use and distribution of naloxone by bystanders and community members in response to suspected opioid overdose. As opioid overdose deaths continue to rise and THN programs continue to expand in Canada, the United States, and Europe, this review will help inform the need for future research and ensure evidence based THN program development and expansion.

## Conclusions

There are several limitations associated with this study. Most of the evidence identified in the systematic reviews relied on observational data. Logistical and ethical issues related to conducting experimental trials in patients at risk of dying from opioid overdoses will likely continue to preclude the establishment of opioid-overdose interventions based on Randomized Controlled Trials (RCT) data. While we attempted to control for quality by limiting our search to studies or documents which used systematic methods to search the literature for evidence related to naloxone, this study did not attempt to provide a synthesis of findings or a quality appraisal of the included literature. Further, our group is based in Canada, and many of the grey literature products identified through targeted websites and expert contacts may be biased towards this region. Scholarly literature searches were also limited to documents in English or French, which may also limit the scope of this study as language of publication limits the geographical range of studies reviewed. Further assessment of included syntheses should be made before they are relied upon for developing recommendations or program amendments.

## Data Availability

All data is publicly accessible through scholarly and grey literature search engines.
